# Molybdenum Speciation and its Impact on Catalytic Activity during Methane Dehydroaromatization in Zeolite ZSM‐5 as Revealed by Operando X‐Ray Methods

**DOI:** 10.1002/anie.201601357

**Published:** 2016-03-17

**Authors:** Inés Lezcano‐González, Ramon Oord, Mauro Rovezzi, Pieter Glatzel, Stanley W. Botchway, Bert M. Weckhuysen, Andrew M. Beale

**Affiliations:** ^1^ Research Complex at HarwellRutherford Appleton LaboratoryDidcotOX11 0FAUK; ^2^ Chemistry DepartmentUniversity College LondonGordon StreetLondonWC1H 0AJUK; ^3^ Inorganic Chemistry and CatalysisDebye Institute for Nanomaterials ScienceUtrecht UniversityUniversiteitsweg 993584CG UtrechtThe Netherlands; ^4^ European Synchrotron Radiation Facility71, Avenue des Martyrs, CS4022038043Grenoble Cedex 9France; ^5^ Central Laser Facility, STFCResearch Complex at HarwellRutherford Appleton LaboratoryDidcotOX11 0QXUK

**Keywords:** heterogeneous catalysis, methane, molybdenum, X-ray techniques, zeolites

## Abstract

Combined high‐resolution fluorescence detection X‐ray absorption near‐edge spectroscopy, X‐ray diffraction, and X‐ray emission spectroscopy have been employed under operando conditions to obtain detailed new insight into the nature of the Mo species on zeolite ZSM‐5 during methane dehydroaromatization. The results show that isolated Mo–oxo species present after calcination are converted by CH_4_ into metastable MoC_*x*_O_*y*_ species, which are primarily responsible for C_2_H_x_/C_3_H_x_ formation. Further carburization leads to MoC_3_ clusters, whose presence coincides with benzene formation. Both sintering of MoC_3_ and accumulation of large hydrocarbons on the external surface, evidenced by fluorescence‐lifetime imaging microscopy, are principally responsible for the decrease in catalytic performance. These results show the importance of controlling Mo speciation to achieve the desired product formation, which has important implications for realizing the impact of CH_4_ as a source for platform chemicals.

The increasing availability of cheap natural gas has attracted growing interest towards direct routes for the conversion of methane into high‐value chemicals.^1^ Catalytic routes that have been investigated include dehydroaromatization, oxidative coupling, and partial oxidation, but are currently not (yet) economically viable.^2^ One of these routes, methane dehydroaromatization (MDA), is particularly promising for the direct conversion of CH_4_ into aromatic compounds and H_2_ using metal‐exchanged zeolites such as Mo/H‐ZSM‐5, since it contains acid sites as well as Mo species possessing dehydrogenation and C−C coupling functionalities.^1^, ^2^, ^3^ It is generally accepted that CH_4_ is activated on the exchanged Mo species, forming C_2_H_4_. Subsequently, C_2_H_4_ reacts on the (remaining) Brønsted acid sites and is converted into aromatic compounds, also leading to coke formation by the consecutive reaction of aromatic derivatives with light olefins.3c, 4 Although active species are proposed to originate from either (MoO_2_)^2+^ monomers or (Mo_2_O_5_)^2+^ dimers,3a,3c, 5 there is also a debate as to whether the active sites are oxidic, carbidic (MoC_*x*_), or oxycarbidic (MoC_*x*_O_*y*_) in nature.3a,3d, 4, 6 Recently, combined UV/Vis absorption and Raman spectroscopies and DFT calculations have shown the formation of monomeric species upon calcination, demonstrating that the debate over the active sites is still ongoing.^7^ In addition, there is no clear understanding of the catalyst deactivation mode, considered to be the main limitation for the commercialization of the process.^1^, ^2^

Herein, we present an operando time‐resolved combined X‐ray diffraction (XRD) and high energy resolution fluorescence detection (K_α_‐detected) X‐ray absorption near‐edge spectroscopy (HERFD‐XANES) study during the MDA reaction on Mo/H‐ZSM‐5. The advantage of using these techniques in combination is that local structure information around the Mo ions can be considered alongside changes in long‐range order, that is, the zeolite framework. Thus any change in catalytic performance can be immediately understood in terms of the structural evolution of the catalyst allowing us to pinpoint active and inactive species, respectively, thereby providing a tenet for future catalyst development. Of particular importance is the application of scarcely used X‐ray emission spectroscopy (XES), which is able to distinguish between ligands surrounding metal ions when they possess a similar atomic number (*Z*), that is, C versus O.^8^ In this work we have used the Mo K_β_ valence‐to‐core (vtc) emission bands (recorded on quenched samples) in conjunction with HERFD‐XANES to be able to unambiguously determine the existence of both MoC_*x*_ and MoC_*x*_O_*y*_ during the course of reaction. Importantly, by measuring under operando conditions we have been able to put this species evolution into the context of the evolving catalytic activity.

Shown in Figure 1 a and Figures S2, 3 in the Supporting Information are the MS data obtained during MDA at 677 °C. For ease of discussion we delineate the MS response into the following time or “event” domains: formation of combustion products, light hydrocarbon evolution, and finally the formation of aromatic compounds. The first responses in time concern the formation of combustion products (Figure S3), that is, CO and CO_2_ in the form of an initial spike from 0–2 min time on stream (TOS) followed by a decrease and plateauing in the detected signal between 2–6 min. This is followed by a second spike between 7–9 min (during this time period the CO response is significantly greater than that of CO_2_) after which the signals for these two components tend towards zero. In this second tranche of combustion products, H_2_O is also detected and exhibits a similar, albeit weaker, response to that of CO. The response for CH_4_ by contrast is observed to climb in intensity up to 7 min (Figure S2), but is followed by a significant decrease and by an upturn in signal intensity between 9–20 min. Between 20–73.5 min the signal for CH_4_ reaches a plateau before gradually decreasing. The final trend in the product profile concerns the aromatic compounds (Figure 1 a), initially detected after 10 min, reaching significant quantities at 30 min followed by a monotonic decrease until the conclusion of the reaction.

**Figure 1:**
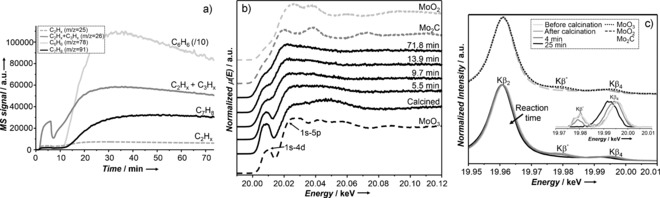
a) MS traces of the products of the MDA reaction. b) Operando Mo K‐edge HERFD‐XANES spectra of Mo/H‐ZSM‐5 acquired after calcination and during the MDA reaction. Dashed lines: spectra of MoO_3_, Mo_2_C, and MoO_2_ reference materials. c) Bottom: K_β_ emission bands (normalized to K_β_ maximum intensity) recorded before and after calcination, and after quenching of the MDA reaction at 4 min and 25 min. Inset: background removed vtc XES. Top: spectra of MoO_3_, Mo_2_C, and MoO_2_ reference materials. Enlarged version shown in Figure S9.

Figure 1 b shows the corresponding Mo K‐edge HERFD‐XANES spectra of Mo/H‐ZSM‐5 zeolite, acquired at the same time as the MS data. Additional HERFD‐XANES data collected under a controlled environment, before and after calcination, and after quenching the reaction at 4, 9, and 73.5 min are presented in Figure S5. All HERFD‐XANES spectra are essentially dominated by a strong pre‐edge peak at 20 008.5 eV attributable to a 1s–4d quadrupole/dipole transition and a 1s–5p dipole transition at 20 025.1 eV followed by a relatively featureless post‐edge region; this is consistent with the presence of Mo species dispersed within the zeolite that do not possess long‐range order. A cursory comparison of the sample obtained after calcination with the spectrum of a Na_2_MO_4_ reference sample,^9^ however, shows a high degree of similarity consistent with the presence of monomeric [MoO_4_]^2−^ species in agreement with the recent study by Gao et al.^7^ We note however, that the presence of small amounts of dimeric Mo–oxo species cannot be readily excluded.

Clear changes in the HERFD‐XANES data were detected during the entire reaction, including a total shift of about 7.7 eV in the rising absorption edge (Figure S6) and a circa 5 % drop in the pre‐edge peak, indicating a reduction/carburization of Mo with TOS. However, we observe that these changes, particularly in the edge position, occur in stages which can be directly linked to the product evolution seen in the MS. The spectrum recorded after 5.5 min of reaction (after the formation of the first set of combustion products) exhibits a comparatively small change in edge position (about 1.5 eV), whereas the edge shift is of the order of 6.0 eV after 9.7 min (after the second spike in combustion products). Finally between 9.7–71.8 min a very small shift of about 0.2 eV is observed (Figures S6,7). These results suggest that Mo reduction/carburization takes place right up until the end of the experiment. The spectrum recorded at 71.8 min, particularly the pre‐edge peak and edge position (20 005.7 eV), is strongly reminiscent of the trigonally coordinated Mo^II^‐containing Mo_2_C structure but without long‐range order. However, to verify this and perhaps more importantly to discriminate between the stages of reduction versus carburization, it is necessary to examine the K_β_ XES data.

K_β_ XES experiments were performed at specific reaction times, the results of which are given in Figure 1 c. As a result of the longer acquisition times required, data were collected on quenched samples (see the Supporting Information). The K_β_ XES spectrum of MoO_3_ was very similar to those of the zeolite‐based samples before and after calcination, with one intense feature at about 19 961 eV attributed to the K_β2_ transition (4p→1s), and two weak bands at higher energies (19 980 and 19 996 eV), corresponding to K_β′′_ and K_β4_ vtc transitions. Although K_β4_ might arise from either 4d to 1s or Mo p density of states to 1s transitions,^8^ K_β′′_ has been suggested to originate from ligand 2s to metal 1s transitions.8b Conversely, the spectrum of MoO_2_ exhibited a decrease in intensity of K_β′′_ (about 70 %), along with a shift to lower energies (about 0.8 eV), whereas K_β′′_ was not detected for Mo_2_C.

Following a similar trend, the spectrum recorded after 4 min of reaction contained a K_β′′_ peak of reduced intensity (about 62 % weaker), which was also shifted in energy (circa 0.7 eV; Figure 1 c). On the basis of previous work which showed that the intensity of K_β′′_ decreases with increasing average Mo−O bond length whereas the energy position decreases with decreasing oxidation state, we propose that at this stage a partial reduction in the Mo oxidation state and a partial replacement of O for C ligands occurs.8b Based on the observations in the K_β_ XES spectra (particularly when considered against the spectra for MoO_3_, MoO_2_, and Mo_2_C), we propose that these results provide compelling evidence for the presence of partially carburized MoC_*x*_O_*y*_ species. From the corresponding HERFD‐XANES data, we estimate an average oxidation state of +5 (Figure S10) which would be consistent with the formation of MoC_*x*_O_*y*_ complexes with a [MoCO](O_fr_)_2_ structure (Scheme 1; O_fr_ indicates framework oxygens). Interestingly, the MS data showed the formation of C_2_H_*x*_/C_3_H_*x*_ at this reaction time, demonstrating that MoC_*x*_O_*y*_ species are also able to activate CH_4_, yielding entirely light hydrocarbons.

**Scheme 1:**
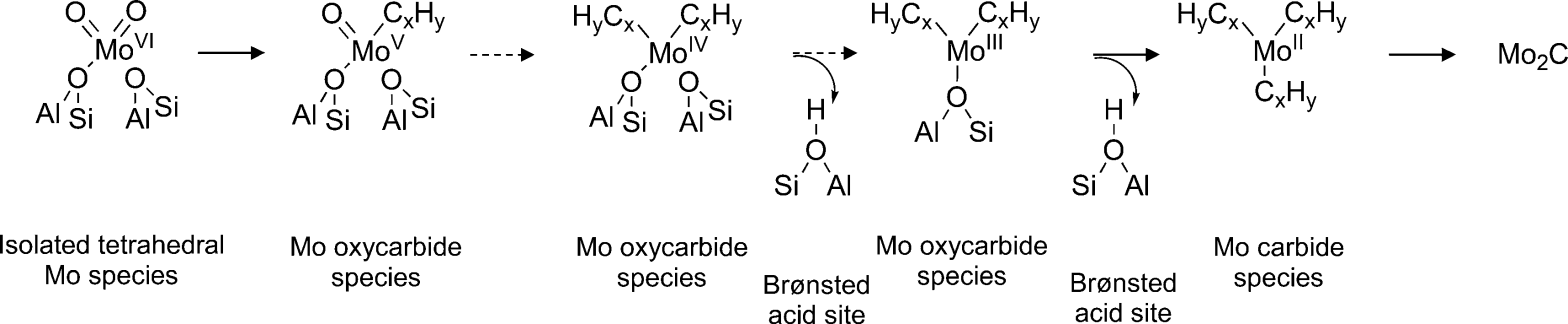
Evolution of Mo species during the MDA reaction on Mo/H‐ZSM‐5 as determined by Mo K‐edge HERFD‐XANES and K_β_ XES.

After 25 min, the K_β′′_ band was not detected, while K_β4_ was further shifted to lower energies (about 3.5 eV; Figure 1 c), resulting in a spectrum very similar to that of Mo_2_C (Figure S13) and indicating the formation of trigonally coordinated MoC_*x*_ (Scheme 1).^10^ The absence of a K_β′′_ peak suggests an absence of Mo–O interactions; on this basis, we conclude that these MoC_*x*_ species are not attached to the zeolite framework. As indicated before, further changes in the HERFD‐XANES were detected between the appearance of aromatic compounds and the end of the reaction, suggesting that Mo centers were not completely reduced or else that reduced Mo species sintered with TOS. Nevertheless, the estimated oxidation state only appears to decrease from +2.1 to +2 between 9 and 73.5 min (Figure S10), suggesting that the changes detected are primarily due to Mo agglomeration rather than further reduction. This is in line with the conclusion that MoC_*x*_ species are not attached to the framework, as well as with previous studies showing the formation of carbide nanoparticles on the external surface, and smaller clusters on the zeolite channels.^11^

Based on the HERFD‐XANES/XES results, a complete pathway for the evolution of the Mo species is given in Scheme 1. Isolated Mo–oxo centers present after calcination are converted into MoC_*x*_O_*y*_ species during the initial contact with CH_4_; these are still attached to the zeolite framework and present varying stoichiometry depending on the extent of the carburization. As carburization of Mo proceeds, MoC_*x*_O_*y*_ species partially detach from the framework, being present as [MoC_3_](O_fr_) complexes. Longer reaction times eventually lead to the transition to a Mo carbide phase, involving the formation of MoC_3_ sites not connected to the framework. As such, these MoC_3_ species are not stable and easily agglomerate with TOS. Ultimately, sintering of MoC_3_ leads to the migration of the clusters to the outer zeolite surface and the formation of larger Mo_2_C‐like nanoparticles.3a, 11a

According to our observations, it is now possible to correlate the type of Mo species and the catalytic behavior. As shown in Figure 1 a, although the maximum benzene formation is only reached after the complete carburization of Mo, MoC_*x*_O_*y*_ species formed at short reaction times are also able to activate CH_4_, as evidenced by the formation of C_2_H_*x*_ and C_3_H_*x*_. It appears, however, that MoC_*x*_ sites are necessary for the formation of aromatic compounds, in agreement with the proposal that MoC_*x*_ species assist in C_2_H_4_ conversion on the Brønsted sites.^12^ In relation to this, the decrease observed in benzene formation after circa 30–35 min may be related to the agglomeration of Mo; sintering leading to a decrease in the active surface area, affecting dehydrogenation where which MoC_3_ species are thought to play a role. Importantly, this could be also due to a decrease in the amount of Brønsted sites (or else a reduced accessibility to these sites).

To further investigate the catalyst deactivation mode, the operando XRD data was next examined (Figure 2 a). The first observation of note is the lack of change in the XRD patterns with increasing TOS, suggesting that no new phases form and that the zeolite ZSM‐5 structure maintains its stability. A closer inspection of the data (Figure S14) also reveals no shift, broadening, or reduction in Bragg reflection intensity, which strongly suggests that neither dealumination (unit‐cell contraction) or else accumulation of carbon within the micropores (lattice expansion) occurs.^13^ However, the catalyst recovered after 73.5 min is black in color, suggesting that if carbon deposition inside the pores does not occur, then it must certainly do so outside on the external zeolite surface.

**Figure 2:**
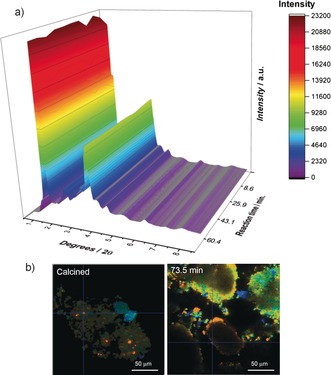
a) Operando XRD data and b) FLIM data of the calcined sample (left) and after 73.5 min of the MDA reaction (right). Scale bars in (b)=50 µm. Species colored from red to blue indicate shorter to longer lived species (see Figures S17 and S19 for further details).

To study the possible build‐up of carbon, fluorescence‐lifetime imaging microscopy (FLIM) measurements were performed on the samples recovered at different reaction times, since fluorescence microscopy alone is unlikely to differentiate between species with similar emission characteristics. As seen in Figures 2 b and Figures S17–19, only scattering species and patches of species with a short fluorescence lifetime (about 150 ps) were detected on the calcined sample, whereas a mixture of both short‐ and long‐lived species (with lifetimes of ns–μs) were detected on the reacted samples, including some particularly long‐lived species that did not decay within the 10 ns FLIM window. As the reaction time increased, the concentration of the very long‐lived species was also found to increase, supporting the idea that fluorescence is emitted from complex carbon species on the external surface, in line with previous reports.^14^ This may be favored by the absence of steric limitations and promoted by the external acid sites and perhaps also suggestive of Al gradients in the zeolite crystals.^15^ The presence of carbon on the external surface will certainly limit accessibility to the Brønsted sites inside the channels, influencing the selectivity towards benzene. Furthermore, from the image at 73.5 min, it can be seen that in addition to being outside of the zeolite pores, the deposited carbon shows a microscale distribution and is located at the periphery of the zeolite particles. Therefore, both sintering of Mo and build‐up of carbon contribute to the gradual deactivation of the catalyst. Note, however, that although steric hindrance would prevent carbon build‐up within the pores, we cannot rule out that this occurs at longer reaction times.

In summary we have shown a novel combination of techniques, coupling for the first time XRD and HERFD‐XANES/XES experiments to investigate, under real operando conditions, the direct conversion of CH_4_ on bifunctional zeolite catalysts. This approach has provided important new insight regarding the need to control, or else maintain, Mo active species to achieve the desired product formation. Although highly transient, the appeal of stabilizing MoC_*x*_O_*y*_ species is that they are highly selective to light hydrocarbons. Although there are issues with stabilizing MoC_*x*_O_*y*_ in the presence of the reaction mixture, it is possible that this could be achieved for example in the presence of co‐fed H_2_O and or O_2_.^16^ MoC_3_ on the other hand are the species to target when we wish to produce aromatic compounds but again either co‐feeding of oxidants to mitigate carbon deposition or else enhancing the interaction with the zeolite is needed for improved stability. Perhaps, however, the most salient observation is that this multi‐technique operando approach has been able to correlate the evolution of active species with distinct reaction products, allowing us to identify clearly the way forward in helping translate the potential of these important catalysts into a reality.

## Supporting information

As a service to our authors and readers, this journal provides supporting information supplied by the authors. Such materials are peer reviewed and may be re‐organized for online delivery, but are not copy‐edited or typeset. Technical support issues arising from supporting information (other than missing files) should be addressed to the authors.

Supplementary
