# Supplementary material for: Molybdenum Speciation and its Impact on Catalytic Activity during Methane Dehydroaromatization in Zeolite ZSM‐5 as Revealed by Operando X‐Ray Methods
Source: Angew Chem Int Ed Engl. 2016 Mar 17;55(17):5215–9. doi: 10.1002/anie.201601357 (PMC5069576; doi:10.1002/anie.201601357)
Supplement: Supplementary file 1 — Supplementary [file ANIE-55-5215-s001.pdf]

Angewandte Chemie
Eine Zeitschrift der Gesellschaft Deutscher Chemiker

Supporting Information

# Molybdenum Speciation and its Impact on Catalytic Activity during Methane Dehydroaromatization in Zeolite ZSM-5 as Revealed by Operando X-Ray Methods

*Inés Lezcano-González, Ramon Oord, Mauro Rovezzi, Pieter Glatzel, Stanley W. Botchway, Bert M. Weckhuysen,* and Andrew M. Beale**

anie_201601357_sm_miscellaneous_information.pdf

# Electronic Supporting Information:

**Table of Contents**


**1. Sample preparation** S-2

**2. Synchrotron studies** S-2

**2.1. Combined XRD and $K_\alpha$-detected HERFD-XANES/XES under operando conditions** S-2

**2.2. HERFD-XANES/ $K_\beta$ XES under controlled environment** S-4

**3. Mass traces of $CH_4$ during MDA reaction** S-5

**4. Mass traces of the products of the induction period** S-6

**5. Operando Mo K-edge HERFD-XANES spectra before and after calcination** S-7

**6. Mo K-edge HERFD-XANES spectra under controlled environment** S-8

**7. Quantification of the changes observed in the operando Mo K-edge HERFD-XANES data** S-9

**8. Mo K-edge HERFD-XANES under operando conditions: 9.7 to 71.8 min of reaction** S-10

**9. $K_\alpha$ emission lines under operando conditions** S-11

**10. $K_\beta$ emission lines under controlled environment** S-12

**11. Correlation Mo K-edge energies with formal oxidation state** S-13

**12. Evidence for the presence of $MoC_xO_y$ structures: Comparison with $MoO_2$ reference** S-15

**13. Comparison with $Mo_2C$** S-17

**14. Operando XRD data** S-18

**15. Ex situ XRD data** S-19

**16. Fluorescence lifetime imaging microscopy** S-20

**References** S-26

## 1. Sample preparation

ZSM-5 zeolite (Si/Al=15) was supplied by Zeolyst International in the ammonium form (CBV3024E), and the H-form of the zeolite material was obtained by calcination in air at 550 ºC (2 ºC.min$^{-1}$ to 120 ºC, held for 30 min; and 5 ºC.min$^{-1}$ to 550 ºC, and 12 h at this temperature). Mo/H-ZSM-5 (4 wt % Mo) was prepared by mixing H-ZSM-5 and $MoO_3$ (Aldrich, 99.95%) powders in an agate mortar for 0.5 h. XRD patterns of as-prepared and calcined Mo/H-ZSM-5 samples were recorded on a Bruker D2 X-ray powder diffractometer equipped with a Co $K_\alpha$ X-ray tube ($\lambda$ = 1.7902 Å).

## 2. Synchrotron studies

### 2.1. Combined XRD and $K_\alpha$-detected HERFD-XANES/XES under operando conditions

XRD/HERFD-XANES/XES studies were performed at ID26 beamline of the European Synchrotron Radiation Facility (ESRF) in Grenoble, France. The electron energy was 6 GeV and the ring current 200 mA. The X-rays were generated by three coupled undulators (U35) working at the fifth harmonic. A cryogenically cooled double-crystal monochromator with Si(111) crystals was used to select the incoming X-ray energy. Mo K-edge HERFD-XANES/Mo $K_\alpha$ XES measurements were recorded in fluorescence mode. The [999] reflection of five Ge(111) spherically bent crystal analyzers (bending radius of 1 m) from the multicrystal spectrometer installed at ID26 was used to select the Mo $K_\alpha$ emission line (approximately 17.4 keV). The total energy resolution of the HERFD-XANES data was estimated to be approximately 4 eV. Energy calibration of the incoming radiation was performed prior to the measurements by recording the K-edge transmission spectrum of a Mo foil and assigning the maximum of the first derivative peak to 20000.0 eV. Background subtraction and normalization were performed using the ATHENA package for the HERFD-XANES data[1] and PyMca[2] for the XES data. XRD data were collected along with the HERFD-XANES/$K_\alpha$ XES measurements. XRD data were acquired using a MAR CCD 165 detector (taper camera) at the X-ray energy of 19900 eV (0.62303 Å). 50 acquisitions of 0.1 s each were used for a pattern. Data were then calibrated and radially integrated using the Datasqueeze software (http://www.datasqueezesoftware.com/).

XRD/HERFD-XANES/XES and catalytic data were acquired simultaneously. A schematic representation of the setup used is shown in Figure S1, together with a photograph. The capillary was connected to a gas manifold system which controlled gas flows by electronic mass flow controllers. The sample was heated by a nitrogen heat gun, allowing heat treatments up to 1000ºC.

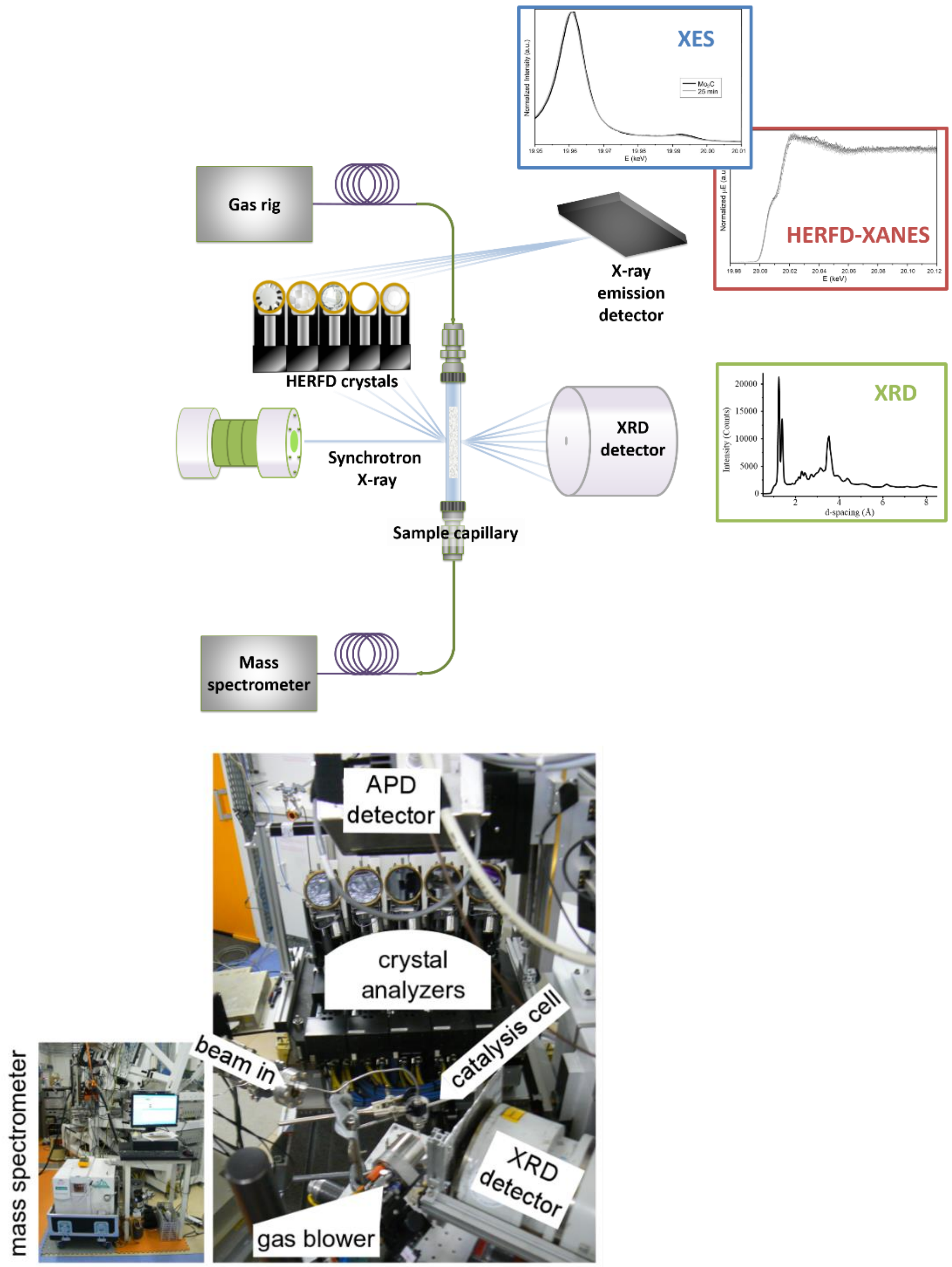


**Figure S1.** Schematic representation of the setup used for performing the combined XRD and HERFD-XANES/XES studies under operando conditions (top), and photograph (bottom).

For the experiments, 100 mg of the as-prepared catalyst (sieve fractions: 0.210-0.125 mm) were placed within a 5 mm quartz capillary, and calcined at 677 ºC for 30 min (20% $O_2$ in He). After flushing with Ar to remove $O_2$, the flowing gas was switched to a $CH_4$/Ar mixture (1:1) and the MDA reaction was carried out at 677 ºC for 73.5 min (GHSV = 1140 $h^{-1}$), very similar to those previously reported in the literature.[3] Catalytic data were recorded using an online mass spectrometer connected to the capillary outlet (Agilent 5975C).

### 2.2. HERFD-XANES/ $K_\beta$ XES under controlled environment

Additional Mo K-edge HERFD-XANES and $K_\beta$ XES measurements were carried out under controlled atmosphere. The spectra were immediately acquired following a fast thermal quench, together with a simultaneous switch of the gas feed to an Ar flow, enabling longer data acquisitions. Reaction times were chosen making use of the operando data, in accordance with the most important spectroscopic events observed. Note that the MDA reaction under the conditions here used is fairly slow, so is reasonable to assume that this methodology enabled to investigate the reaction at the same conditions as the operando data. Indeed, the catalyst bed temperature rapidly decreased after removing the heating source, being below 600 °C after less than 10 s.

HERFD-XANES data were acquired as described in section 2.1, though several successive scans were recorded and averaged in order to improve the data quality. For the Mo $K_\beta$ XES mesurements, the incident beam energy was set at 20100 eV, and the spectra were recorded with an array of five Si(1,1,0) spherically bent crystal analyzers (bending radius of 1 m) using the [12, 12, 0] reflection. The total energy bandwidth in the X-ray emission detection was approximately 5 eV. An avalanche photodiode (APD) was used as a detector. The $K_\beta$ main and satellite lines were normalized to $K_\beta$ maximum intensity.

### 3. Mass traces of $CH_4$ during MDA reaction

As seen in Figure S2, methane consumption was monitored using three different fragments (m/z = 13, 15 and 16), and the trends observed were identical. Based on the similarity in the catalyst preparation/reaction conditions used here with those previously reported we estimate that a maximum $CH_4$ conversion of 10.2% was reached at 677 ◦C after the initial activation period.[3]

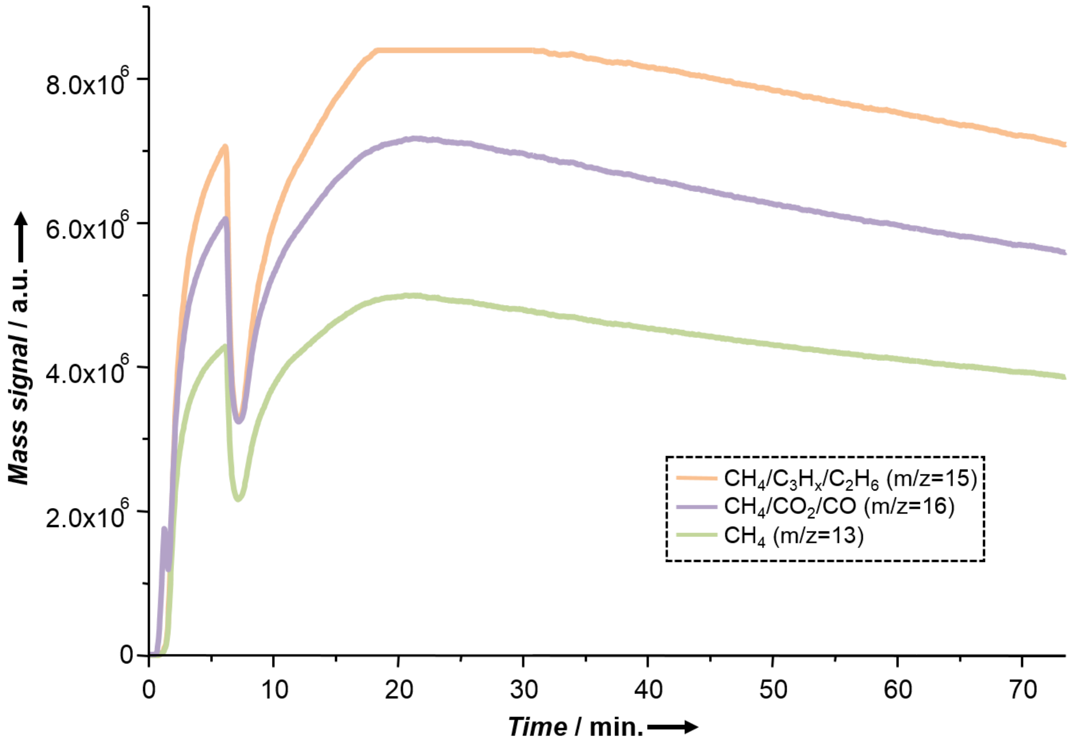


**Figure S2.** Mass traces of $CH_4$ during the MDA reaction on Mo/H-ZSM-5 (4 wt. %, Si/Al=15) at 677 °C for 73.5 min ($CH_4$/Ar =1).

### 4. Mass traces of the products of the induction period

Mass traces of $CH_4$, and CO, $CO_2$ and $H_2O$, i.e. the main products of the induction period, were followed during the course of the MDA reaction (Figure S3). Note however that formation of $H_2$ was not monitored.

For the discussion of the data we assume that the *m/z* peak at 28 is mainly due to CO, while that at 44 is due to $CO_2$, since these are present at higher concentrations than $C_2H_x$ and $C_3H_8$ at short reaction times.

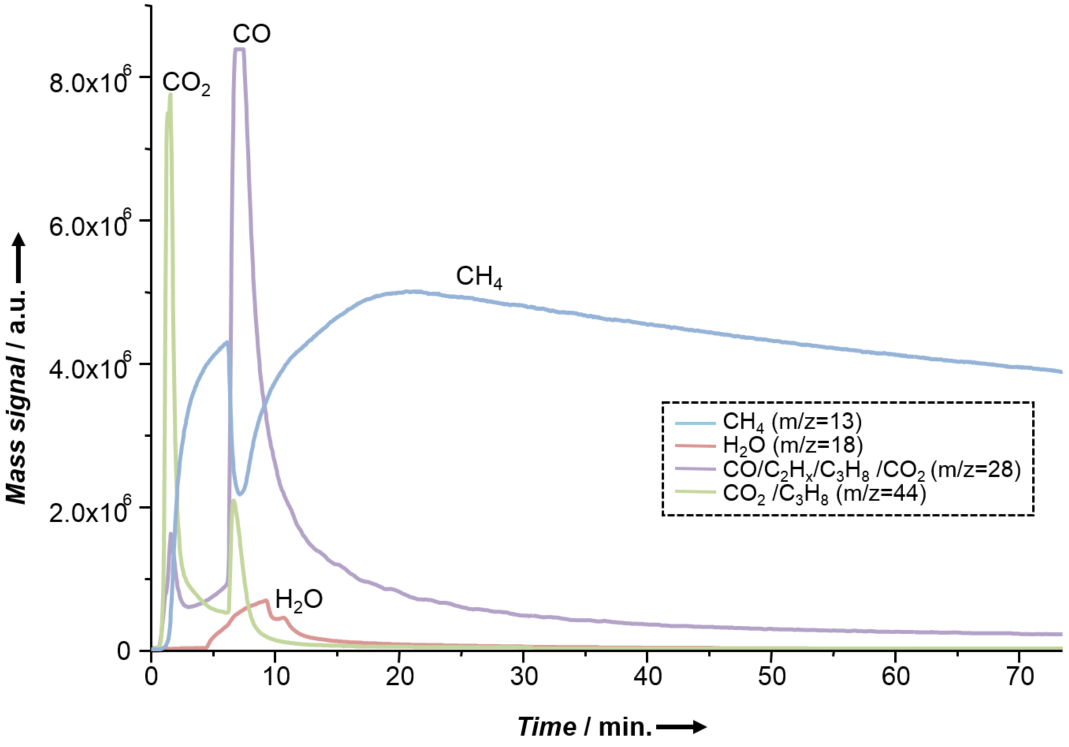


**Figure S3.** Mass traces of $CH_4$ and the reaction products formed during the induction period of the MDA reaction on Mo/H-ZSM-5 (4 wt. %, Si/Al=15) at 677 °C for 73.5 min ($CH_4$/Ar =1).

**5. Operando Mo K-edge HERFD-XANES spectra before and after calcination**

Figure S4 shows the Mo K-edge HERFD-XANES spectra of Mo/H-ZSM-5 zeolite, acquired in operando conditions before and after calcination at 677 °C, together with the spectrum of a $MoO_3$ reference. The Mo K-edge HERFD-XANES spectrum of the fresh catalyst is very similar to that for crystalline $MoO_3$, with octahedral coordination. Calcination led however, to a gradual edge shift of -0.3 eV, along with an increase in the pre-edge peak intensity (~ 20 %), consistent with a change from octahedral to tetrahedral coordination.[3-4] As reflected in the relative amplitudes of the multiple scattering post-edge features, Mo sites present after calcination do not have a long range order and exist as highly dispersed species within the zeolite. Furthermore, while there is a very good agreement between the $MoO_3$ reference and the fresh catalyst, the calcined Mo/H-ZSM-5 zeolite is not matched as good, supporting the conclusion that Mo species are highly dispersed upon calcination.

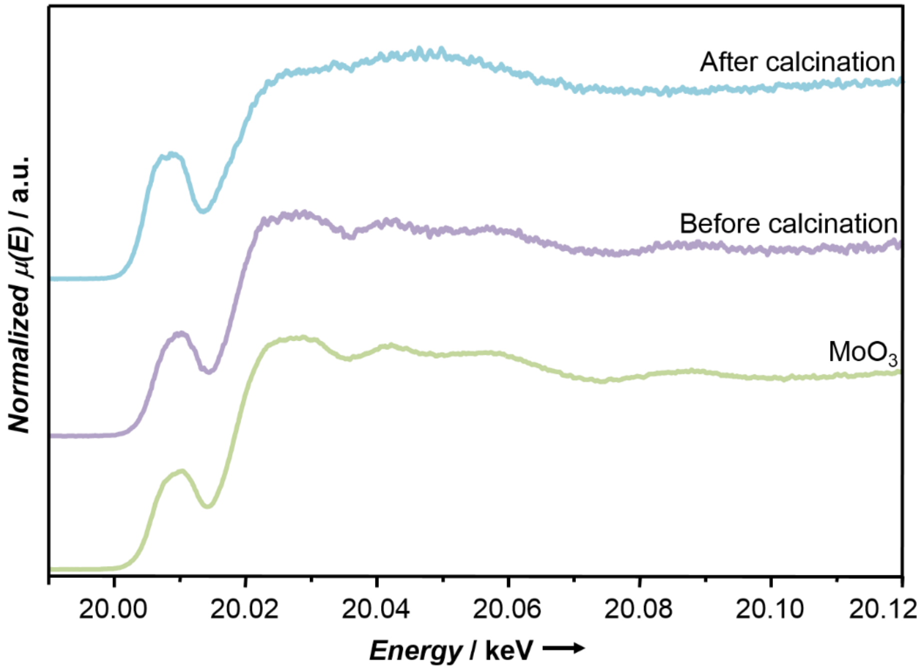


**Figure S4.** Operando Mo K-edge HERFD-XANES spectra of Mo/H-ZSM-5 acquired before and after calcination.

## 6. Mo K-edge HERFD-XANES spectra under controlled environment

As described in section 2.2, complementary Mo K-edge HERFD-XANES spectra were acquired just after a fast thermal quench (below 600ºC after less than 10 s), together with a simultaneous switch of the gas feed to an Ar flow, enabling longer data acquisitions. As seen in Figure S5, the spectral features and the changes observed with time are consistent with the operando data (Figures 1b and S4), giving further support to the approach here used.

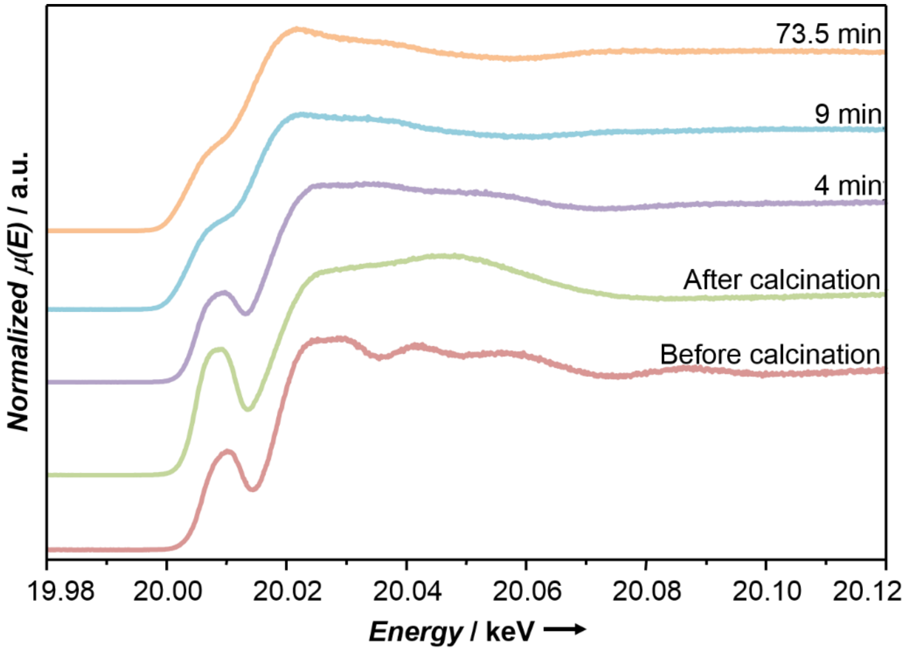


**Figure S5.** Mo K-edge HERFD-XANES spectra of Mo/H-ZSM-5 before and after calcination, and after quenching the MDA reaction (677 °C; $CH_4$/Ar =1) at 4, 9 and 73.5 min.

## 7. Quantification of the changes observed in the operando Mo K-edge HERFD-XANES data

Figure S6 shows the variation in the edge position during the MDA reaction (determined from the HERFD-XANES data under controlled atmosphere (Figure S5) as the energy position at half-step height).

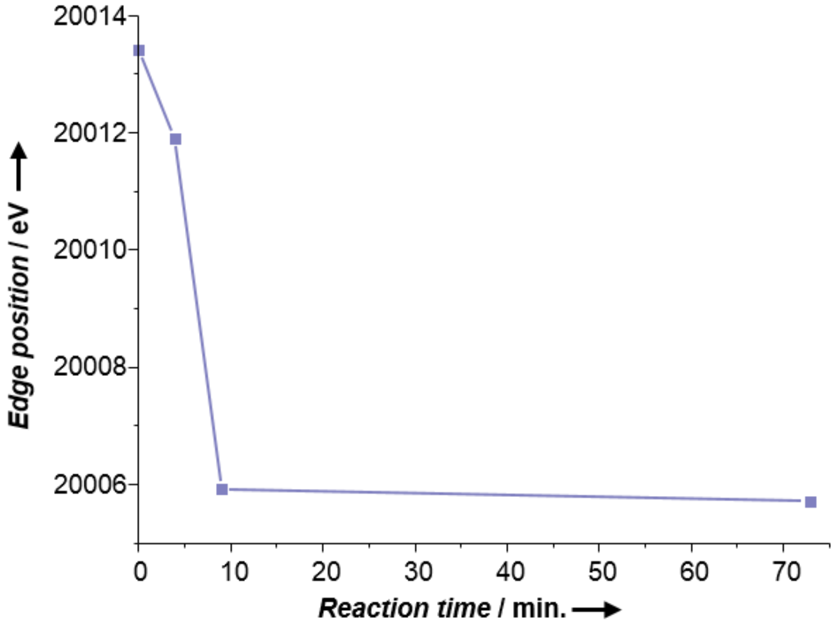


**Figure S6.** Variation in the edge position (determined as the energy position at half-step height) during the MDA reaction (677 °C; $CH_4$/Ar =1) on Mo/H-ZSM-5.

## 8. Mo K-edge HERFD-XANES under operando conditions: 9.7 to 71.8 min of reaction

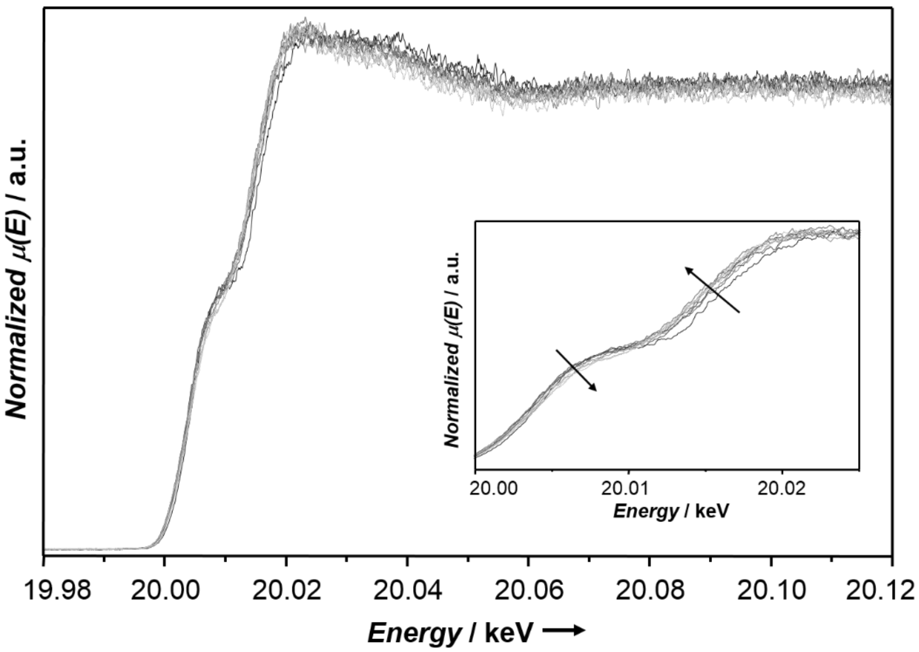


**Figure S7.** Operando Mo K-edge HERFD-XANES spectra acquired between 9.7 and 71.8 min of the MDA reaction at 677 °C ($CH_4$/Ar =1) on Mo/H-ZSM-5 (4 wt. %, Si/Al=15).

## 9. $K_\alpha$ emission lines under operando conditions

In line with the operando HERFD-XANES data, a change in the oxidation state and geometry of Mo was observed by operando $K_\alpha$ XES - acquired simultaneously during the MDA reaction, as reflected by a shift of the emission line to higher energies (see Figure S8). The same trend was seen for the reference compounds used, for which the energy position increased in the order: $MoO_3$< $MoO_2$<$Mo_2C$, indicating the gradual removal of non-zeolitic oxygen atoms and the reduction of Mo during the early stages of the reaction.

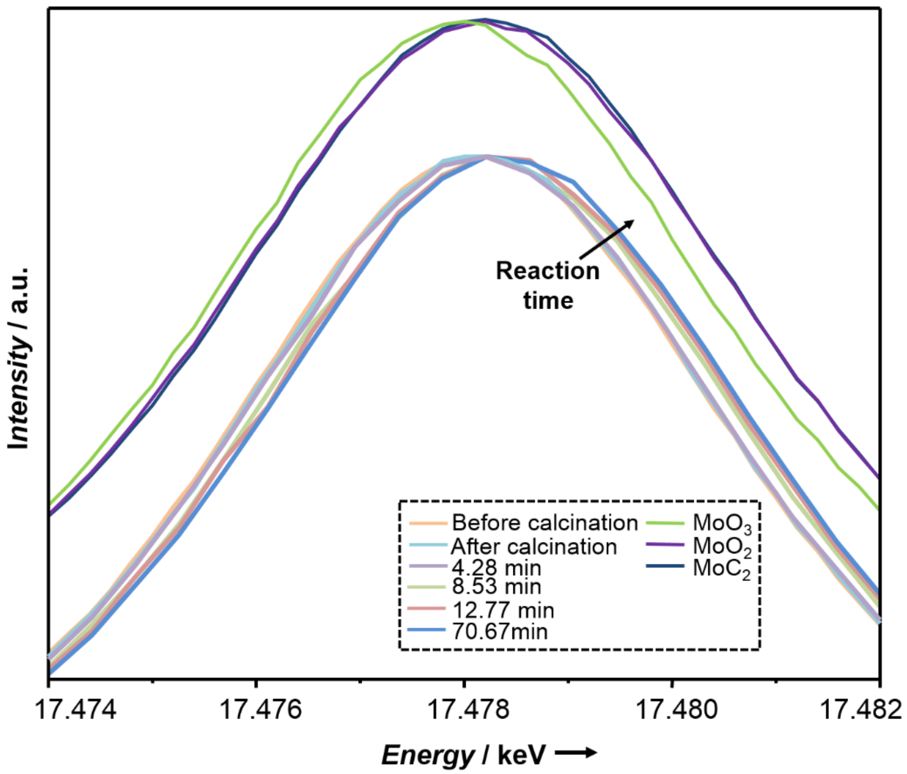


**Figure S8.** $K_\alpha$ emission lines (normalized by the integrated spectral intensity) acquired before and after calcination, and during MDA reaction at 677 °C ($CH_4$/Ar =1) on Mo/H-ZSM-5 for 4.28, 8.53, 12.77, and 70.67 min. Spectra of $MoO_3$, $Mo_2C$ and $MoO_2$ reference compounds are also included.

**10. $K_\beta$ emission lines under controlled environment**

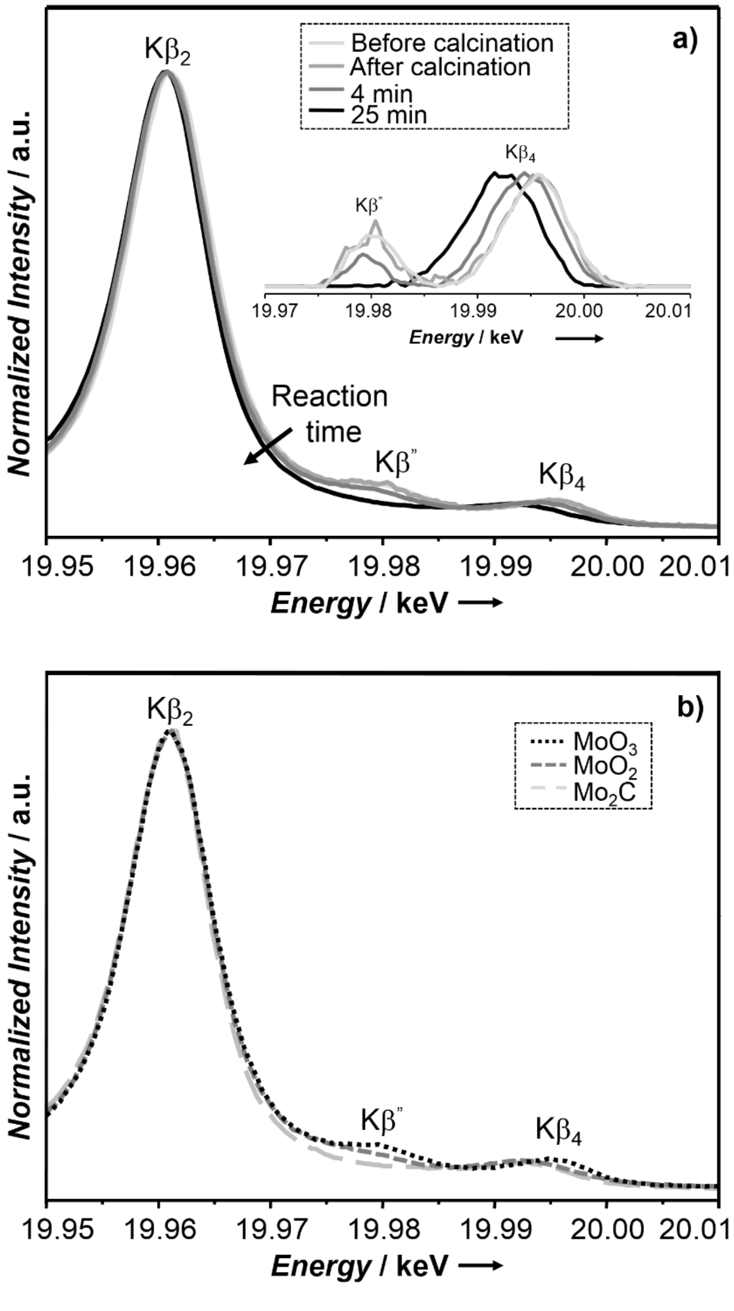


**Figure S9.** a) $K_\beta$ emission lines (normalized to $K_\beta$ maximum intensity) recorded before and after calcination, and after quenching the MDA reaction at 4 and 25 min. Background removed vtc XES is reported in the inset; b) spectra of $MoO_3$, $Mo_2C$ and $MoO_2$ references.

## 11. Correlation Mo K-edge energies with formal oxidation state

The evolution of Mo oxidation state during reaction was etimated by correlating Mo K-edge energies with oxidation state. K-edge energies were determined from the HERFD-XANES data under controlled atmosphere (Figure S5), as the energy position at half-step height.[5]

First, Mo K-edge energies versus formal oxidation state for Mo reference compounds ($MoO_3$, $MoO_2$ and $Mo_2C$; see Table S1) were fitted by least-squares regression. The fitted line had a $R^2$ value of 0.995. Mo oxidation state of the catalyst during activation and MDA reaction was then predicted from the linear relation found (see Figure S10 and Table S2).

**Table S1.** Oxidation state and Mo K-edge energies (determined from the HERFD-XANES data as the energy position at half-step height) for Mo reference compounds.

| Sample | Oxidation state | K-edge energy (eV) |
|---|---|---|
| $MoO_3$ | 6 | 20013.7 |
| $MoO_2$ | 4 | 20010.1 |
| $Mo_2C$ | 2 | 20005.5 |

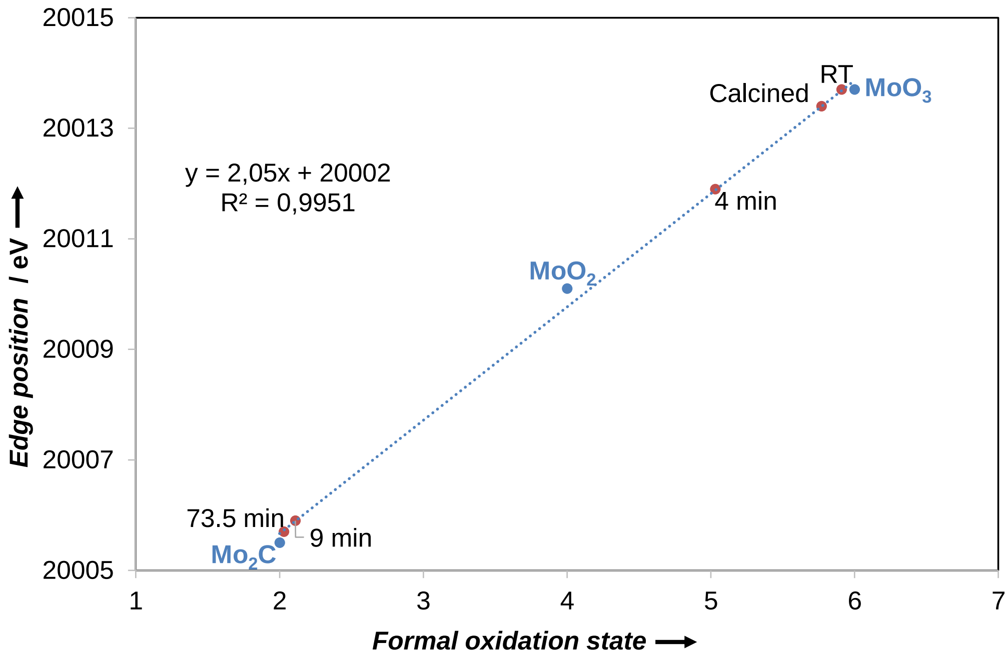


**Figure S10.** Evolution of Mo oxidation state during activation and MDA reaction predicted from the linear relation found for Mo reference compounds (Mo K-edge energy = 2.05 x Mo oxidation state + 20001.57; $R^2$ = 0.995).

**Table S2.** Evolution of Mo oxidation state during activation and MDA reaction predicted from the linear relation found for Mo reference compounds (Mo K-edge energy = 2.05 x Mo oxidation state + 20001.57; $R^2$ = 0.995).

| **Sample** | **Oxidation state** | **K-edge energy (eV)** |
|---|---|---|
| Before calcination | 5.9 | 20013.7 |
| After calcination | 5.8 | 20013.4 |
| 4 min of reaction | 5.0 | 20011.9 |
| 9 min of reaction | 2.1 | 20005.9 |
| 73.5 min of reaction | 2.0 | 20005.7 |

As expected, the oxidation number of the zeolite sample before and after calcination was almost +6, very close to that of $MoO_3$. Just after $CH_4$ initial contact, a marked decrease in oxidation state is seen, being +2.1 after 9 min of exposure. In contrast, increasing reaction times lead only to a very slight decrease in the oxidation number.

## 12. Evidence for the presence of $MoC_xO_y$ structures: Comparison with $MoO_2$ reference

Comparison of the operando HERFD-XANES spectrum after 5.5 min of reaction with that of $MoO_2$ (Figure S11) evidences that *the species present are not* $MoO_2$.

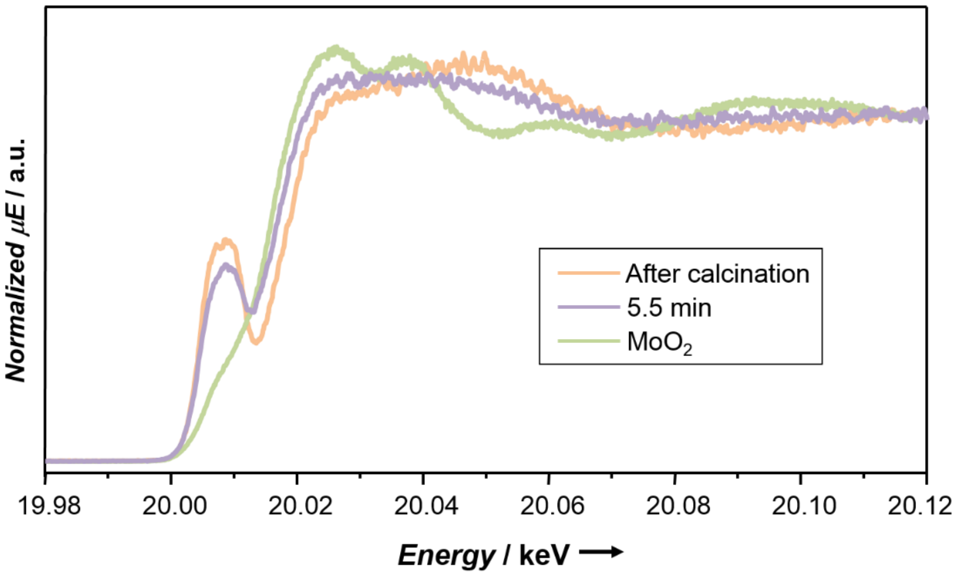


**Figure S11.** Mo K-edge HERFD-XANES spectra of Mo/H-ZSM-5, after calcination and 5.5 min of the MDA reaction, and $MoO_2$ reference.

Comparison of the $K_\beta$ XES spectra acquired after 4 min of the MDA reaction with those recorded after calcination and for a $MoO_2$ reference (Figure S12) indicates that *the presence of a mixture of Mo-exchanged species and $MoO_2$ after 4 min of reaction can be ruled out*, as two distinctive $K_{\beta 4}$ peaks would be observed in the spectrum.

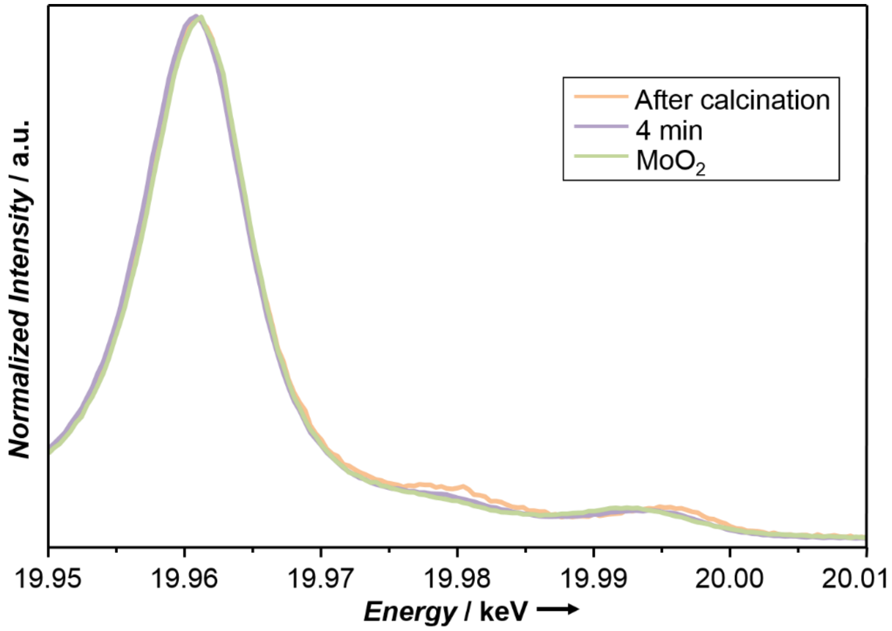


**Figure S12.** $K_\beta$ emission lines (normalized to $K_\beta$ maximum intensity) of Mo/H-ZSM-5, after calcination and 4 min of the MDA reaction, and $MoO_2$ reference.

### 13. Comparison with $Mo_2C$

Figure S13 compares the $K_\beta$ XES spectra acquired after 25 min of the MDA reaction with that recorded for a $Mo_2C$ reference. $K_\beta$ emission spectrum showed the disappearance of the $K_{\beta''}$ band for either $Mo_2C$ or the zeolite sample. We note however, that the spectral resolution is much lower for 4d than for 3d transition metals, so it is also possible that this band could not be resolved.

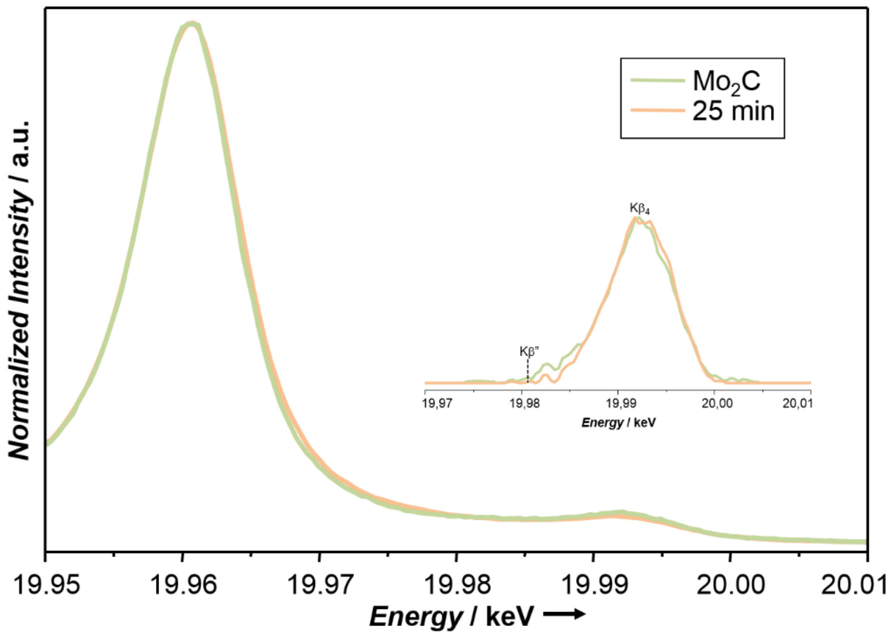


**Figure S13.** $K_\beta$ emission lines (normalized to $K_\beta$ maximum intensity) of Mo/H-ZSM-5 after 25 min of the MDA reaction, and $Mo_2C$ reference. Background removed vtc XES (normalized to $K_{\beta 4}$) is reported in the inset.

## 14. Operando XRD data

Figure S14 shows a comparison between the operando XRD data recorded after 7 and 70 min of the MDA reaction

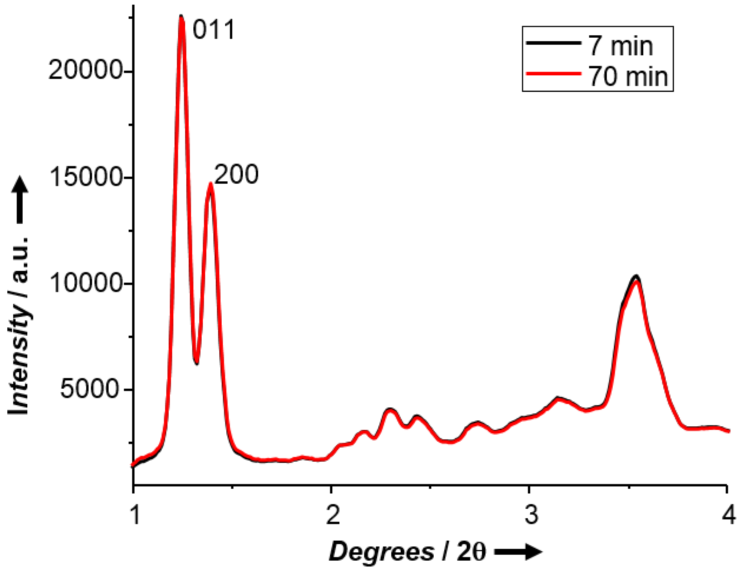


**Figure S14.** Operando XRD data of Mo/H-ZSM-5 zeolite (4 wt.% Mo; Si/Al=15) after 7 and 70 min of the MDA reaction.

**15. Ex situ XRD data**

In agreement with the operando XRD data (Figure 2, top), the ex situ XRD patterns acquired on the samples recovered at different reaction times (Figure S15) showed no peak shift or broadening, indicating that the zeolite structure was maintained under reaction conditions. Moreover, the absence of extra peaks indicates that no large $MoO_3$ crystallites were present after calcination, and that not $Mo_2C$ or $Al_2(MO_4)$ large clusters were formed during reaction.

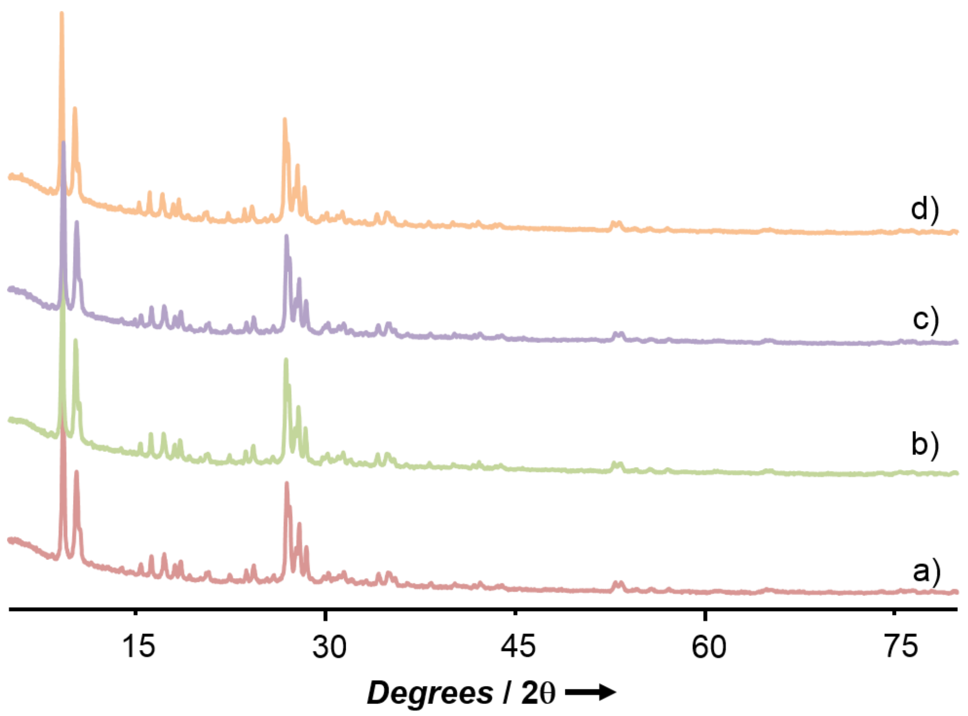


**Figure S15.** Ex situ XRD data of Mo/H-ZSM-5 zeolite (4 wt.% Mo; Si/Al=15) after calcination (a), and after MDA reaction (677 ºC, $CH_4$/Ar =1) carried out for 9 min (b), 25 min (c), and 73.5 min (d).

## 16. Fluorescence lifetime imaging microscopy

Fluorescence-lifetime imaging microscopy (FLIM) is an imaging technique, which produces an image based on the excited state decay time per pixel from a fluorescent sample. The contrast is generated by the differences in the lifetime of individual fluorophores, so the pseudo-color of each pixel is determined by the fluorescence lifetime. As seen in Figure S16, when a molecule absorbs a photon it enters an excited state and from this state it can return to the ground state by emitting a photon. The fluorescence lifetime is a measure of the time a molecule stays in the excited state before returning to the ground state.[6] The excited state therefore reports directly on the presence of different chromophores as well as the environment of the excited electron.

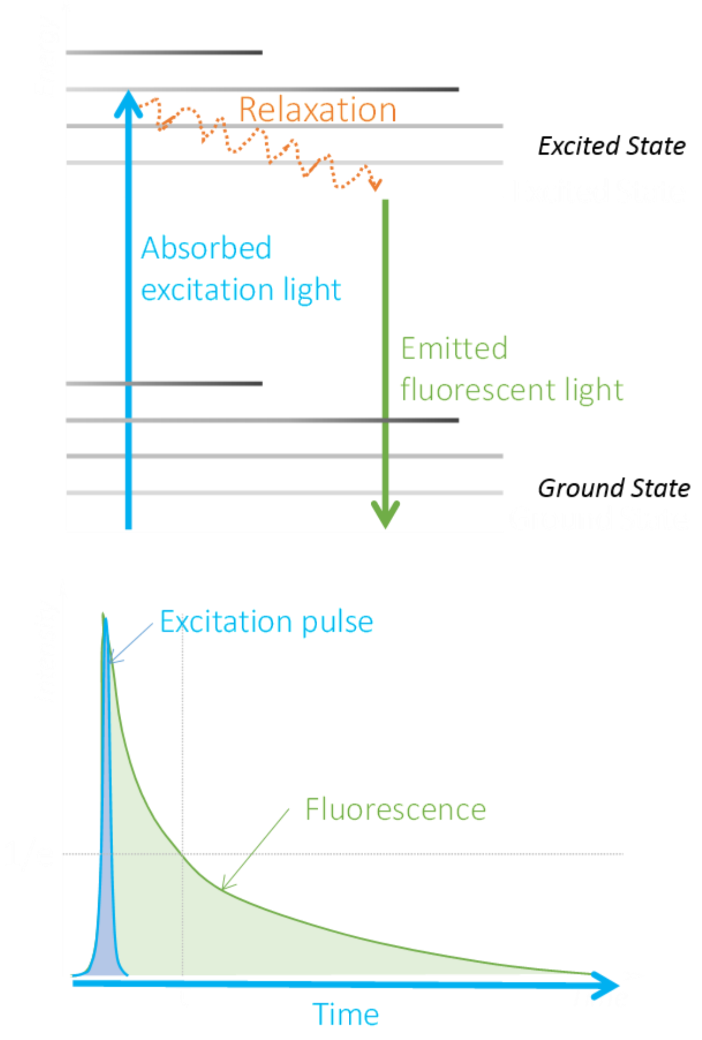


**Figure S16.** Principle of fluorescence-lifetime imaging microscopy from a modified Jabloski diagram.

Multiphoton excitation was employed to access possible UV and UV absorbing chromophore. The multiphoton photon excitation experiments were performed at the Rutherford Appleton Laboratory as described by Botchway et al.[7] Briefly a mode locked Mira titanium sapphire laser (Coherent Lasers Ltd, USA), generating 180 fs pulses at 75 MHz and emitting light at a wavelength of 720-970nm nm was used for the multiphoton photon excitation. The laser was pumped by a solid state continuous wave 532 nm laser (Verdi V18, Coherent Laser Ltd), with the oscillator fundamental output of 765 ± 5 nm. The laser beam was focused to a diffraction limited spot through a water immersion ultraviolet corrected objective (Nikon VC x60, NA1.2) and samples illuminated at the microscope stage of a modified Nikon TE2000-U with UV transmitting optics. The focused laser spot was raster scanned using an XY galvanometer (GSI Lumonics) or a modified Nikon EC1 confocal scanhead to allow multiphoton microscopy (SWB ref). Fluorescence emission was collected without de-scanning, bypassing the scanning system and passed through a coloured glass (BG39) filter. The scan was operated in normal mode and line, frame and pixel clock signals were generated and synchronized with an external fast microchannel plate photomultiplier tube used as the detector (R3809-U, Hamamatsu, Japan). These were linked via a Time-Correlated Single Photon Counting (TCSPC) PC module SPC830. All multiphoton and FLIM images are acquired at 212 μm full field of view. Lifetime calculations were obtained using SPCImage analysis software Version 5.2 (Becker and Hickl, Germany) and using equation below.

$$F(t) = a_1 e^{-t/\tau_1} + a_2 e^{-t/\tau_2} + a_3 e^{-t/\tau_3}$$

Where *F(t)* represents intensity or photon counts at time t. $a_1$ is a normalization term (the pre-exponential factor) and $\tau$ is the lifetime. $a_1$, $a_2$ *and* $a_3$ represents multiple exponential decay components as would be expected in such system as the treated zeolite.

Figure S17 shows the FLIM data of Mo/H-ZSM-5 after calcination. In this sample the decay lifetime is mainly characteristic of that observed for a scattering material (ps within the instrument response function ~50 ps), although some limited ns species were observed. Origins of these are unknown and are currently under investigation.

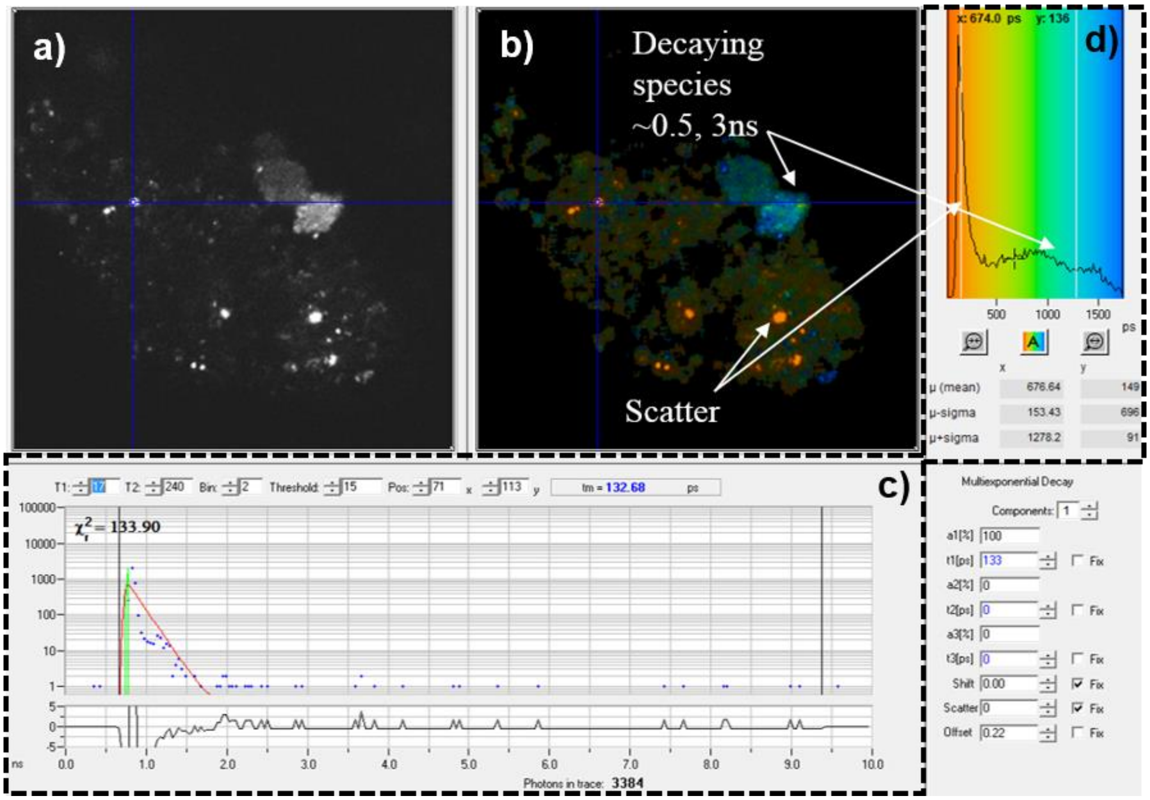


**Figure S17.** FLIM data of Mo/H-ZSM-5 after calcination. a) microscope image, b) FLIM image, c) decay at a representative xy pixel, and d) rainbow color map representing the distribution of all pixels in the image and the lifetime spread.

Figure S18 shows the FLIM data of Mo/H-ZSM-5 after 9 min of the MDA reaction. The results obtained indicate the presence of higher amounts of the longer lived species (i.e. lifetime of ns, including 100 ns-lived species) together with the scattering material which also increased.

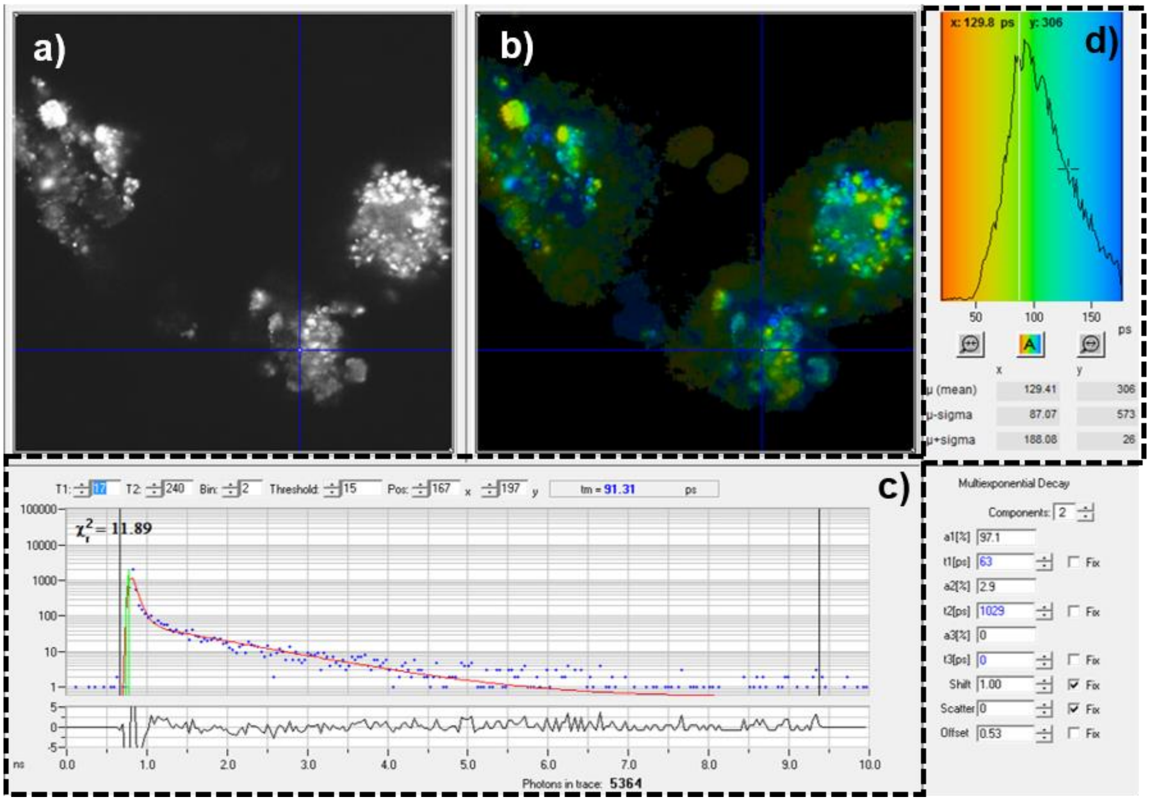


**Figure S18.** FLIM data of Mo/H-ZSM-5 after 9 min of the MDA reaction. a) microscope image, b) FLIM image, c) decay at a representative xy pixel, and d) rainbow color map representing the distribution of all pixels in the image and the lifetime spread.

Figure S19 shows the FLIM data of Mo/H-ZSM-5 after 73.5 min of the MDA reaction. This sample showed a mixture of excited state lifetime with an overall significant increase of the emitting species over that at 9 min of reaction.

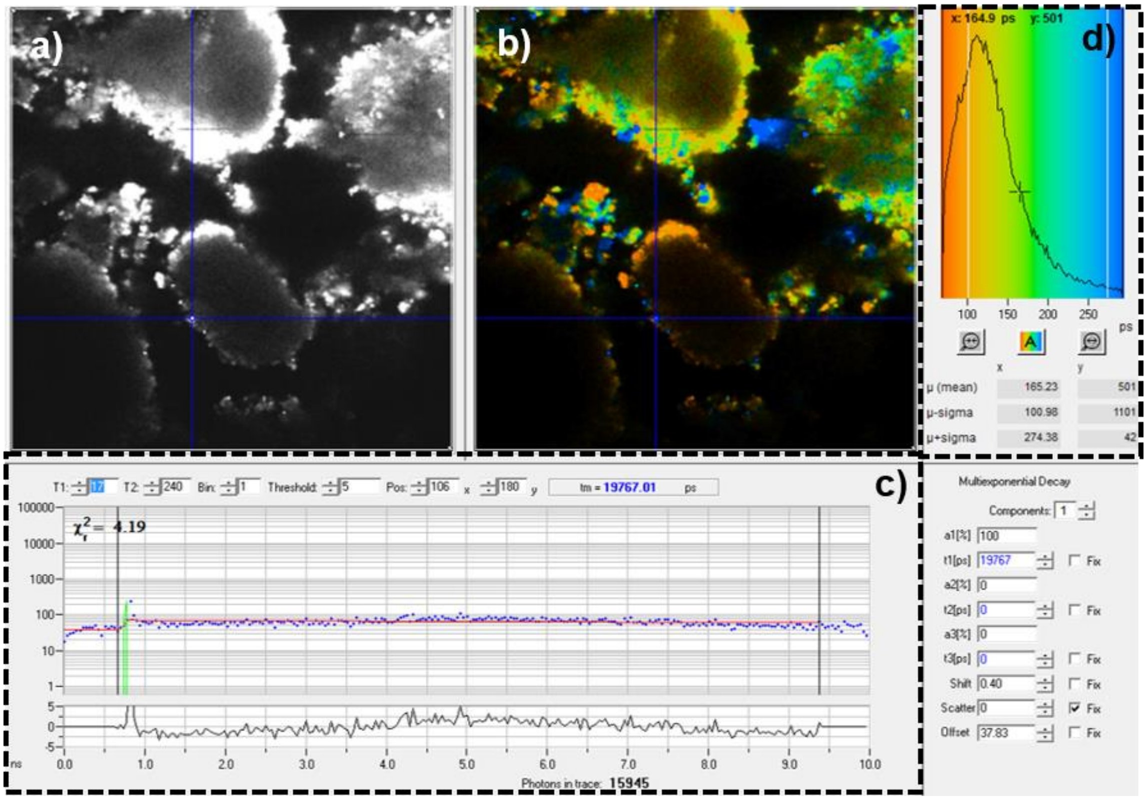


**Figure S19.** FLIM data of Mo/H-ZSM-5 after 73.5 min of the MDA reaction. a) microscope image, b) FLIM image, c) decay at a representative xy pixel, and d) rainbow color map representing the distribution of all pixels in the image and the lifetime spread.

Figure S20 shows the FLIM data of $Mo_2C$ reference. Note that only scattering species were detected in the sample, giving further support to the assignment of the very long-lived species seen in the reacted samples to the presence of growing amounts of carbon. No clear discernible correlation was measured in the lifetime window. Image shows only photon detection at the detector but with poor correlation with the excitation pulse.

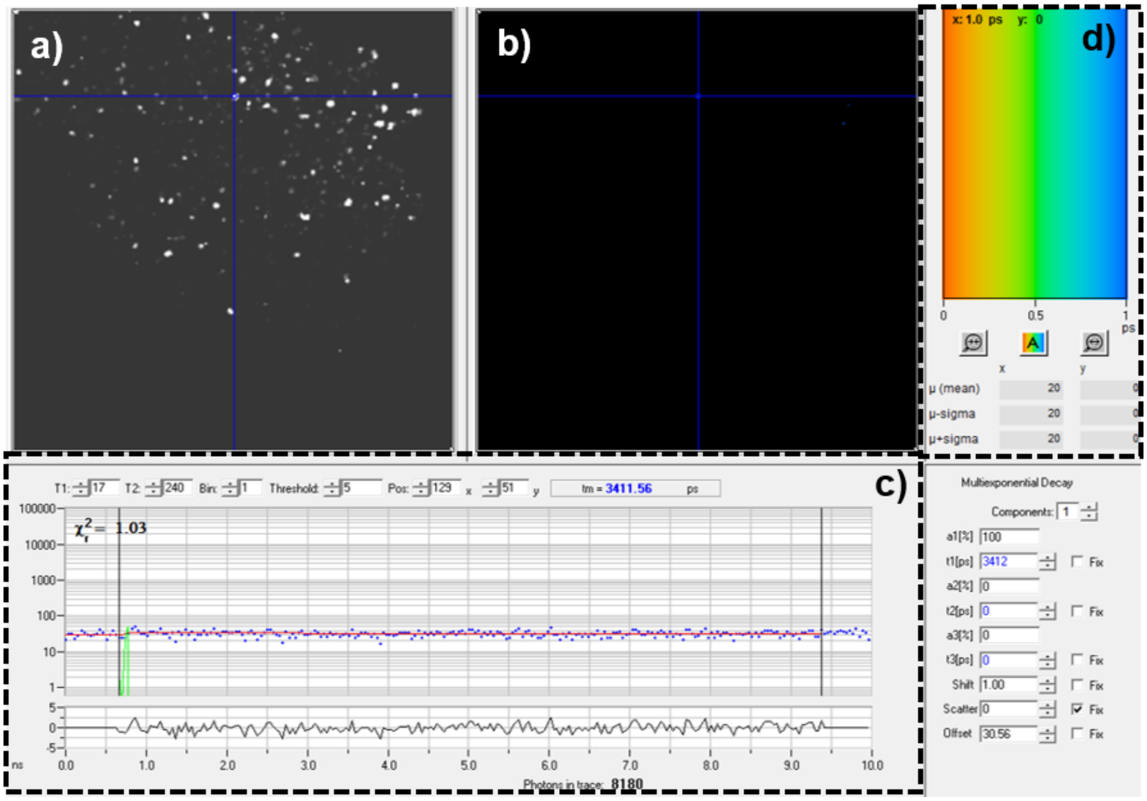


**Figure S20.** FLIM data of $Mo_2C$ reference. a) microscope image, b) FLIM image, c) decay at a representative xy pixel, and d) rainbow color map representing the distribution of all pixels in the image and the lifetime spread.

## References


[1] B. Ravel, M. Newville, *J. Synchrotron Rad.* **2005**, *12*, 537.

[2] V.A. Solé, E. Papillon, M. Cotte, Ph. Walter, J. Susini, *Spectrochim. Acta Part B*, **2007**, *62*, 63.

[3] R. W. Borry, Y. H. Kim, A. Huffsmith, J. A. Reimer, E. Iglesia, *J. Phys. Chem. B* **1999**, *103*, 5787.

[4] W. Li, G. D. Meitzner, R. W. Borry III, E. Iglesia, *J. Catal.* **2000**, *191*, 373; J. P. Thielemann, T. Ressler, A. Walter, G. Tzolova-Müller, C. Hess, *Appl. Catal. A: Gen.* **2011**, *399*, 28; N. Ohler, A. T. Bell, J. Phys. Chem. B **2005**, *109*, 23419.

[5] L. Li, M. R. Morrill, H.; Shou, D. G. Barton, D. Ferrari, R. J. Davis, P. K. Agrawal, C. W. Jones, D. S. Sholl, *J. Phys. Chem. C* **2013**, *117*, 2769.

[6] W. Becker, *J. Microsc.* **2012**, *247*, 119.

[7] S. W. Botchway, K. M. Scherer, S. Hook, C. D. Stubbs, E. Weston, R. H. Bisby, A. W. Parker, *J. Microsc.* **2015**, *258*, 68.


Note – processed Mo K-edge XANES data, X-ray emission data and powder diffraction asci files can be found at:

https://rcahdrive.rc-harwell.ac.uk/index.php/s/mr7vqRCWcphIFUC?path=%2F201601357
